# Rituximab for Early‐Onset Juvenile Dermatomyositis Complicated by Interstitial Lung Disease

**DOI:** 10.1155/crpe/9776022

**Published:** 2025-12-22

**Authors:** M. Olszewska, E. Mężyk, M. Kurbiel, W. Kmiecik, D. Turowska-Heydel, M. Sobczyk, Z. Żuber

**Affiliations:** ^1^ Department of Paediatrics, Jagiellonian University Medical College, Cracow, Poland, cm-uj.krakow.pl; ^2^ Clinical Department of Paediatrics and Rheumatology, St. Louis Regional Specialized Children’s Hospital, Cracow, Poland; ^3^ Department of Paediatrics, Faculty of Medicine, Andrzej Frycz Modrzewski Cracow University, Cracow, Poland, ka.edu.pl

**Keywords:** case report, interstitial lung disease, juvenile dermatomyositis, rituximab

## Abstract

Juvenile dermatomyositis (JDM) is the most common inflammatory myopathy in children. Among its various multisystem manifestations, JDM‐associated interstitial lung disease (JDM‐ILD) can cause significant disability and mortality. Data on the management of JDM‐ILD are limited. However, rituximab can be an effective and safe option not only for refractory cases but also for initial treatment. We report the case of a three‐year‐old girl with JDM‐ILD. The patient had a four‐month history of progressive muscle weakness and rash. On admission, she presented with major skin involvement and decreased muscle strength. Laboratory tests revealed mild normocytic anemia and elevated lactate dehydrogenase levels. She was positive for Antitranscription intermediary factor 1–gamma antibody (TIF1‐γ). Whole‐body magnetic resonance imaging confirmed typical muscle involvement. High‐resolution computed tomography of the chest revealed JDM‐ILD with a nonspecific interstitial pneumonia pattern, affecting approximately 30% of the lung volume. The treatment included methylprednisolone pulses, intravenous immunoglobulins, and methotrexate. Due to evidence of severe disease with a high risk of mortality, rituximab was also administered. The treatment was well tolerated. During follow‐up visits, normalization of muscle strength and reduction in skin and lung involvement were observed. As demonstrated in the presented patient, rituximab can be considered an early intervention in cases of severe, life‐threatening manifestations of JDM, such as JDM‐ILD. Importantly, the treatment was not related to any significant adverse events.

## 1. Introduction

Juvenile dermatomyositis (JDM) is the most common subtype of idiopathic inflammatory myopathies (IIMs) in children. Its incidence is very low, estimated at 2‐3 cases per million annually [1, 2]. JDM typically affects children aged 5 to 10 years, with a female predominance [3, 4]. However, a younger age at first clinical presentation is observed in approximately 25% of patients. JDM is a systemic capillary vasculopathy that causes symmetric proximal muscle weakness and characteristic skin manifestations. However, the gastrointestinal, cardiac, and respiratory systems can also be involved. Although interstitial lung disease (ILD) in JDM patients is rare, its occurrence can significantly increase disability and mortality [5, 6]. In adults with IIM, the preferred treatment for ILD is a combination of glucocorticoids with one of the following: mycophenolate, azathioprine, rituximab, or calcineurin inhibitor [7]. Unlike in the adult population, there are no specific recommendations for the treatment of JDM‐associated ILD (JDM‐ILD) in children.

## 2. Case Presentation

A three‐year‐old girl was admitted to the hospital because of progressive muscle weakness and a skin rash. Four months before admission, her parents noticed periungual erythema on the girl’s fingers. About a month later, she developed facial swelling along with a photosensitive, erythematous rash and red papules on the extensor surfaces of the metacarpophalangeal (MCP) and proximal interphalangeal (PIP) joints. At the same time, the girl limited her usual daily activities, such as riding a scooter and going for walks. She experienced difficulties in climbing stairs. One month before admission, she was unable to squat and get up from a supine position without assistance. Two weeks later, she developed axillary ulcerations without previous trauma. Her past medical history was unremarkable, including recent infections. She has a family history of vitiligo and asthma in her first‐degree relatives.

**Figure 1:**
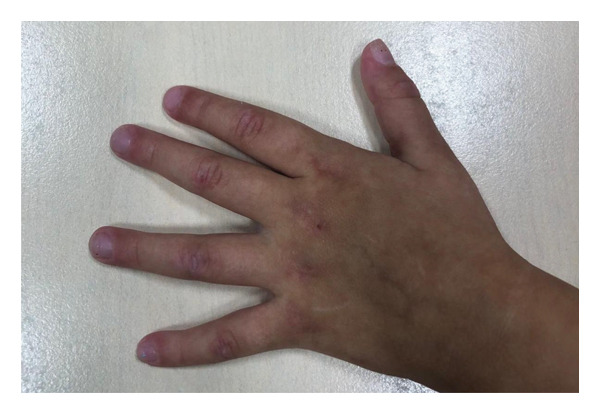
Gottron papules on the extensor surface of the MCP and PIP joints.

**Figure 2:**
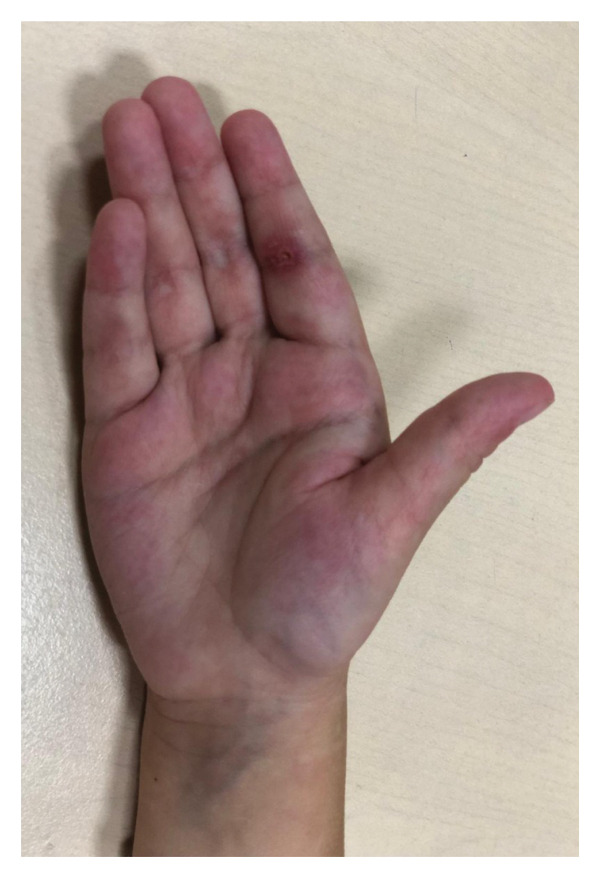
Hypopigmentation and scarring on the palmar surface of PIP joints.

**Figure 3:**
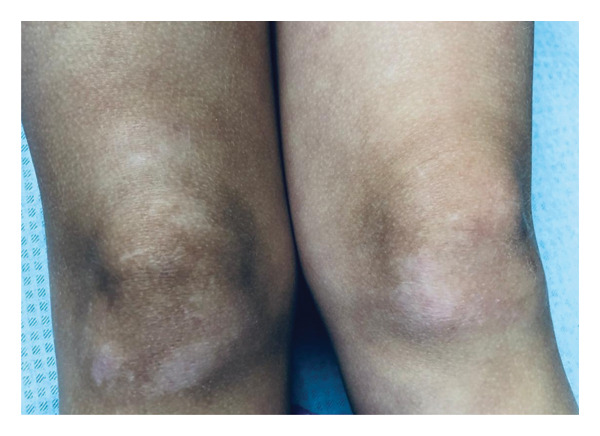
Depigmentation of the skin on the extensor side of the knee joints.

**Figure 4:**
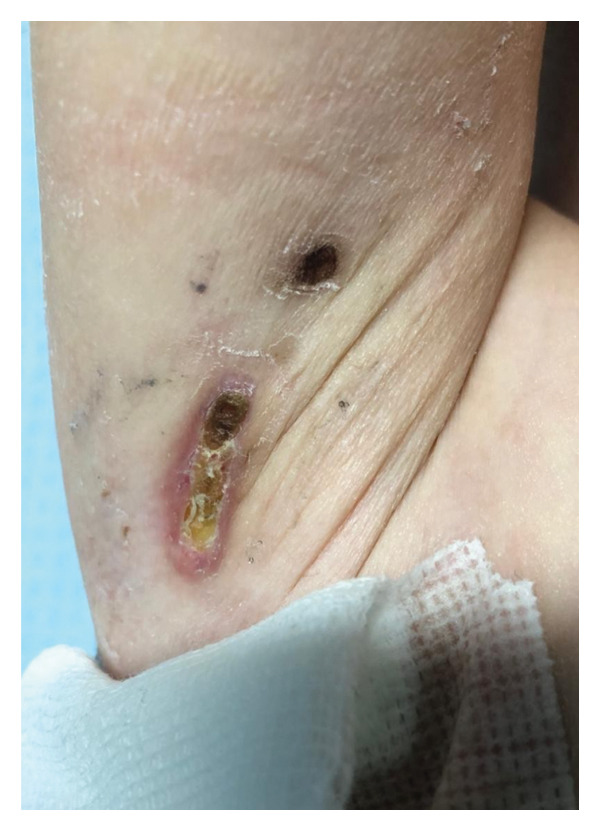
Right axillary ulceration.

**Figure 5:**
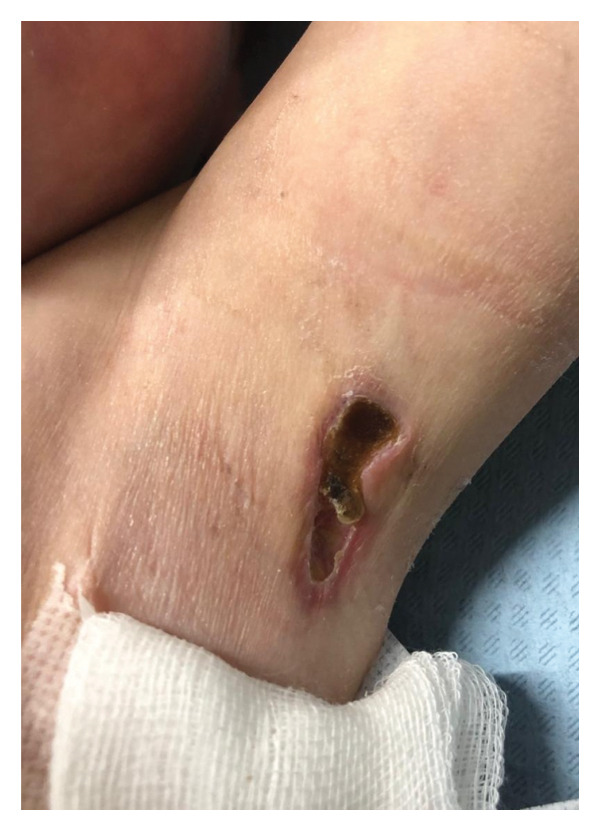
Left axillary ulceration.

**Figure 6:**
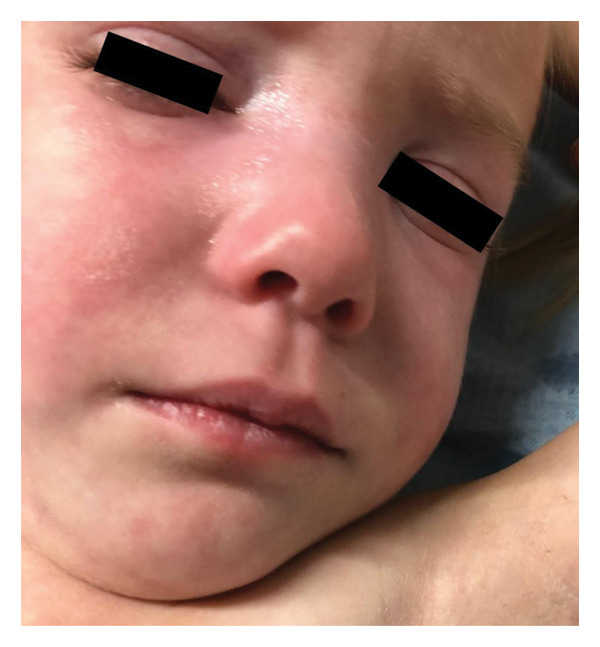
Malar erythema, swollen cheeks, and upper eyelids.

On admission, she presented with a heliotropic rash, malar erythema, swollen cheeks and upper eyelids, Gottron papules on the extensor surfaces of the MCP and PIP joints, hyperpigmentation and scarring on the palmar surface of the PIP joints, Gottron sign on the elbows, depigmentation of the skin on the extensor side of the knee joints, bilateral deep axillary ulcerations, and livedo reticularis on the extremities and trunk (Figures 1, 2, 3, 4, 5, and 6). Her muscle strength was severely decreased in the proximal and axial muscles. She did not present with dysphagia or dysphonia. Due to severely limited spontaneous physical activity, the presence of dyspnea on exertion could not be assessed, but it was not observed at rest. During the first days of hospitalization, she additionally developed an ulcer on the upper eyelid.

The laboratory test results revealed a normal erythrocyte sedimentation rate (ESR) and C‐reactive protein, mild normocytic anemia, and elevated lactate dehydrogenase at 330 U/L (192–321 U/L). Levels of creatine kinase, aldolase, and transaminase were within normal limits. The EUROLINE autoimmune inflammatory myopathies 16‐antigen strip (EUROIMMUN AG, Lubeck, Germany) was strongly positive for antitranscription intermediary factor 1–gamma antibody (TIF1‐γ) and negative for other antibodies.

**Figure 7:**
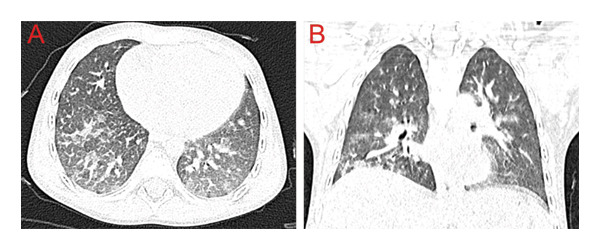
HRCT showing nonspecific interstitial pneumonia‐bilateral ground‐glass opacities and reticular changes with peripheral and basal predominance. (A) Transverse scan. (B) Coronal scan.

The nailfold capillaroscopy revealed significant microcirculation changes, including the predominance of avascular areas and single megacapillaries. Whole‐body magnetic resonance imaging (MRI) showed edema and atrophy of the shoulder and pelvic girdle muscles. The muscle biopsy was not performed. According to the EULAR/ACR Classification Criteria for Idiopathic Inflammatory Myopathies, she was classified as a definite case of IIM [8]. The Childhood Myositis Assessment Scale (CMAS) was estimated at 20/52 points. The Cutaneous Assessment Tool (CAT) Activity Score was 8/17 points. Because of the patient’s young age, which limited the possibility of pulmonary function testing, and concerning high disease activity, she underwent high‐resolution computed tomography (HRCT) of the chest. The imaging revealed bilateral ground‐glass opacities and reticular changes affecting 30% of the lung volume. JDM‐ILD of a nonspecific interstitial pneumonia pattern was diagnosed (Figure 7).

A combination therapy involving five methylprednisolone pulses at 20 mg/kg/day and intravenous immunoglobulins (IVIG) at 2 g/kg was started, followed by a regimen of oral methylprednisolone at 0.5 mg/kg/day and methotrexate. Due to evidence of severe disease with a high risk of mortality, a single dose of rituximab at 375 mg/m^2^ was administered. The drug infusion was well tolerated, but it was complicated by a mild and transient increase in transaminase levels. At discharge, the patient was prescribed prophylaxis with trimethoprim–sulfamethoxazole.

At the follow‐up examination one month later, significant improvement in muscle strength and resolution of skin lesions were observed. The patient did not develop any new symptoms of JDM, nor did she experience side effects related to treatment. The CMAS was estimated at 33/52 points, while the CAT Activity Score decreased to 3/17 points. Laboratory tests showed that the patient was no longer anemic and the LDH level normalized. During hospitalization, she received IVIG and the second dose of rituximab at 375 mg/m^2^. A mild increase in transaminase levels was observed again following rituximab infusion. After discharge, the methylprednisolone dose was gradually reduced.

Following the next month, the patient’s muscle strength and skin condition continued to improve, as reflected in the CMAS assessment of 48/52 points and the CAT Activity Score of 2/17 points. Six months after the initial diagnosis, HRCT of the chest showed regression of lung involvement, while disease activity in the muscular and cutaneous domains remained low. Due to transient hypogammaglobulinaemia, the third dose of rituximab at 375 mg/m^2^, initially scheduled at six months, was postponed and administered at 10 months without complications. One year after the initial diagnosis, she remained in good condition and continued treatment with methotrexate, IVIG, and methylprednisolone 4 mg/day.

## 3. Discussion

We reported a case of early‐onset JDM complicated by ILD. According to recommendations, the girl can be classified as a high‐risk patient because of skin ulcerations and lung involvement [6]. Although the frequency of JDM‐ILD seems to be lower than in adults with IIM, its presence can significantly affect prognosis as it is associated with patients’ mortality [9].

There are marked discrepancies in the incidence of JDM‐ILD across different studies. In the Japanese cohort, it was found in over 30% of patients, whereas in the United States and Denmark, it was observed in only 5% and 8%, respectively [5, 10, 11]. However, the observed difference may not only be related to ethnicity‐specific factors but also to a different diagnostic approach. It should be emphasized that, similar to our patient, JDM‐ILD is most often asymptomatic itself, or symptoms are scarcely observed because of general muscle weakness. Therefore, the Single Hub and Access point for pediatric Rheumatology in Europe (SHARE) recommends routine pulmonary function tests for all children at the time of diagnosis [6]. However, their usefulness is limited in young children. Therefore, the true incidence of JDM‐ILD may be underestimated if chest HRCT is not performed. Conversely, in Japan, the Scientific Research Group for Pediatric Rheumatic Diseases (SRGPRD) recommends screening for JDM‐ILD by HRCT in every patient at diagnosis [12]. These differences in clinical guidelines can lead to an underestimation of JDM‐ILD incidence in European cohorts. The most frequent pattern of JDM‐ILD is nonspecific interstitial pneumonia, which was also observed in our patient [13].

The risk factors for developing JDM‐ILD have not been clearly identified. However, in adults with IIM, positivity for antisynthetase, Antimelanoma differentiation–association gene 5 (MDA‐5), anti‐Ku, anti‐Pm/Scl, and anti‐Ro52 antibodies, as well as mechanic’s hands, arthritis or arthralgia, and ulcerating lesions, were associated with an increased risk of ILD [7]. Some of these factors are suggested to be significant in the pediatric population. In a retrospective analysis of Japanese and Chinese cohorts of juvenile IIM patients, anti‐MDA‐5 antibody was found to be a risk factor for ILD [10, 14]. On the contrary, no relationship was found between myositis‐specific antibodies and JDM‐ILD in the Danish patients. However, the analysis included only 4 patients with JDM‐ILD (5). Higher ESR and interleukin 10 levels were also postulated as risk factors in the pediatric population [15]. Importantly, the observations above are based only on retrospective analysis, and there is a lack of prospective studies on JDM‐ILD. To date, there are no data regarding the relationship between the age of JDM diagnosis and the development of ILD.

The TIF1‐γ antibody is frequently detected in JDM, particularly in Caucasians and young children [14]. As with our patient, TIF1‐γ positivity is associated with severe cutaneous involvement, including ulcerations. However, the risk of JDM‐ILD is not increased in patients positive for this antibody [16].

Since the risk factors for JDM‐ILD are not clearly defined, all children should undergo assessment for lung involvement at the time of JDM diagnosis, even if no specific symptoms are present [6, 12]. Due to age‐related limitations of pulmonary function tests, performing HRCT of the chest might be necessary in younger children. However, due to the risk of radiation, studies on JDM‐ILD biomarkers are an area of growing interest. It is postulated that serum Krebs von den Lungen‐6 (KL‐6), a mucin‐like protein produced by Type 2 alveolar lung cells, may become a marker of JDM‐ILD, as its level correlates with the severity and extent of lung involvement on HRCT [17]. The usefulness of this biomarker has been previously confirmed in adults with connective tissue disease–associated ILD [18].

The low incidence of JDM limits the possibility of conducting randomized controlled trials (RCTs) to determine optimal therapeutic interventions. To date, only four RCTs analyzing pharmacological treatment have been conducted, and none targeted JDM‐ILD [19–22]. There are also no guidelines dedicated exclusively to JDM‐ILD management. However, in cases of severe disease, such as major organ involvement, SHARE recommends a combination of glucocorticoids, methotrexate, and cyclophosphamide. In comparison, the SRGPRD strategy for treating JDM‐ILD includes glucocorticoids with calcineurin inhibitor or cyclophosphamide [6, 12]. Importantly, high cumulative doses of cyclophosphamide can cause permanent infertility in both women and men [23]. Therefore, as rituximab is not known to interfere with fertility, it was preferred over cyclophosphamide in our patient [24].

The rationale for targeting B cells in JDM treatment results from the well‐documented role of B cells in the disease’s immunopathogenesis. In muscle biopsies from JDM patients, B cells infiltration has been observed in the perivascular regions [25]. Furthermore, high serum concentrations of B‐cell–activating factor have been reported in patients with dermatomyositis [26]. Not only do B cells produce pathogenic autoantibodies but also present antigens to T cells and release proinflammatory cytokines. Hence, they play a crucial role in the initiation and maintenance of inflammation in JDM. To date, rituximab treatment in JDM‐ILD has been reported only in single cases [27–30]. Among them, three patients were treated successfully, and one died. Only one patient was TIF1‐γ positive and had a good response to rituximab, but unlike in our case, he was clinically symptomatic for JDM‐ILD [30]. More data are available for adults. He et al., in a systematic review on ILD related to dermatomyositis, reported an over 70% response rate to rituximab treatment [31].

Another promising therapeutic options in JDM‐ILD are Janus kinase (JAK) inhibitors. In patients with refractory JDM and coexisting JDM‐ILD, Zhang et al. reported successful treatment with tofacitinib in 60% of cases, whereas Wang et al. showed improvement during baricitinib therapy in 50% [32, 33].

In the presented case, the early initiation of rituximab can be questioned, as it is only considered in refractory cases of JDM according to available recommendations [6, 12]. Although our patient was negative for MDA‐5 antibody, we assume that early rituximab treatment was justified by the high mortality risk related to advanced JDM‐ILD involving 30% of lung volume and severe cutaneous presentation. A similar therapeutic approach was described in another case of TIF1‐γ–positive JDM‐ILD [31].

In conclusion, we report a case of early‐onset JDM with severe cutaneous manifestations and advanced ILD. Importantly, our patient did not present any clinical symptoms of lung involvement on admission and was MDA‐5 negative. At the current level of knowledge, it indicates the need for routine diagnostic testing for JDM‐ILD even in asymptomatic patients without known risk factors. The latest recommendations place rituximab as a therapeutic option for refractory symptoms. However, we postulate that it should also be considered an early intervention in cases of severe, life‐threatening disease. In our patient, the combination of rituximab, methotrexate, IVIG, and glucocorticoids was successful in treating the cutaneous, muscle, and lung domains. Significantly, we did not observe any serious side effects. However, to optimize management and reduce mortality, RCTs on JDM‐ILD are urgently needed.

## Consent

Written consent has been obtained from the patient’s legal guardian for publication purposes.

## Conflicts of Interest

The authors declare no conflicts of interest.

## Funding

No specific funding was obtained for this study.

## Data Availability

The data that support the findings of this study are available from the corresponding author upon reasonable request.
